# Draft Genome Sequences of Four Antibiotic-Resistant *Salmonella* Strains Isolated from Australian Red Meat Animal Species

**DOI:** 10.1128/MRA.00925-19

**Published:** 2019-08-29

**Authors:** Amreeta Sarjit, Joshua T. Ravensdale, Ranil Coorey, Narelle Fegan, Gary A. Dykes

**Affiliations:** a School of Public Health, Curtin University, Bentley, Western Australia, Australia; b School of Molecular and Life Sciences, Curtin University, Bentley, Western Australia, Australia; c CSIRO Agriculture and Food, Brisbane, Queensland, Australia; University of Rochester School of Medicine and Dentistry

## Abstract

The genome sequences of four antibiotic-resistant Salmonella strains isolated from red meat animals in Australia are presented. Multidrug-resistant Salmonella enterica serovar Heidelberg 329 and Salmonella enterica serovar Typhimurium 2470 harbored an IncHI2 plasmid similar to the multidrug-resistant *S.* Heidelberg strain N13-01290 plasmid pN13-01290_23 previously isolated in Canada.

## ANNOUNCEMENT

Salmonella enterica is a leading cause of human foodborne bacterial gastroenteritis worldwide (1). The widespread use of antibiotics in animal husbandry has resulted in multiantibiotic-resistant *Salmonella* strains in many countries, but their presence in Australia is generally low (2).

Forty-nine *Salmonella* strains consisting of Salmonella enterica serovars Anatum, Bovismorbificans, Heidelberg, Newport, Saintpaul, Typhimurium, and Virchow, isolated from different red meat animals consisting of cattle, sheep, and goats in Australia from 2001 to 2013, were isolated as described previously (3–5). All strains were grown in tryptone soy broth (TSB; Oxoid, UK) at 37°C for 24 h for genomic DNA extraction. Genomic DNA was extracted and purified using the Wizard genomic DNA purification kit (Promega, USA), according to the manufacturer’s instructions. The purity and concentration of the genomic DNA were determined using agarose gel electrophoresis and by a Qubit 2.0 fluorometer. The genomic DNA was fragmented and tagged using a Nextera XT DNA library preparation version 3 600-cycle MiSeq kit, followed by whole-genome sequencing on an Illumina MiSeq V3 sequencer (2 × 300-bp reads). The quality of the reads was determined using the BayesHammer error correction tool to improve the quality of the assembly. Genomes were assembled using SPAdes, and the genome qualities of the final assemblies were confirmed using the Quality Assessment Tool (QUAST) for genome assembly algorithm, followed by annotation using Rapid Annotations using Subsystems Technology (RAST) and the NCBI Prokaryotic Genome Annotation Pipeline (PGAP) (6–8). Antimicrobial resistance genes were determined using ResFinder (9). The genome sequences of three strains of *S.* Heidelberg (329 and 632 from goat rumen and 2581 from cattle feces) and one strain of *S.* Typhimurium (2470 from cattle feces), which were the only strains found to have antibiotic resistance genes, are presented here. Plasmid replicons were determined using PlasmidFinder (10). Plasmid contigs were identified using a local BLAST search against a database of known plasmids in PATRIC and mlplasmids (11, 12). Default parameters were used for all software, unless otherwise specified.

Genome features, including the total number of sequence reads, number of contigs, *N*_50_, *L*_50_, assembly size, GC content, and numbers of coding sequences (CDS), tRNAs, rRNAs, and plasmids across the four *Salmonella* genomes are shown in Table 1. The sources and dates of isolation of the four *Salmonella* strains are also shown in Table 1. A 100% orthologous average nucleotide identity was identified between the genomes of *S.* Heidelberg 329 and *S.* Typhimurium 2470 and between *S.* Heidelberg 632 and 2581, using the OrthoANI algorithm (13). This suggests a close phylogenetic relationship between the two sets of strains.

**TABLE 1:** Summary of the whole-genome features of *S.* Heidelberg and *S.* Typhimurium strains

Parameter	Data for strain:			
	*S*. Heidelberg 329	*S*. Heidelberg 632	*S*. Heidelberg 2581^*a*^	*S*. Typhimurium 2470
Source	Goat rumen	Goat rumen	Cattle feces	Cattle feces
Date of isolation (day/mo/yr)	13/12/2001	27/2/2002	27/8/2013	26/2/2013
Total no. of sequence reads	3,047,918	1,395,904	1,851,866	1,790,304
No. of contigs	69	34	28	63
*N*_50_ (bp)	191,631	740,277	641,189	231,719
*L*_50_	9	2	2	7
Assembly length (bp)	5,069,302	4,780,505	4,685,585	5,071,508
GC content (%)	51.9	52.1	52.2	51.9
No. of CDS	5,172	4,773	4,654	5,181
No. of tRNAs	81	79	79	82
No. of rRNAs	12	13	13	11
No. of plasmids	3	3	0	2
Accession no.	SWJR01000000	SWMX00000000	SWMW00000000	SWMV00000000
Contig accession no. (plasmid)	SWJR01000007 (p329_1), SWJR01000047 (p329_2), SWJR01000048 (p329_3)	SWMX01000015 (p632_1), SWMX01000017 (p632_2), SWMX01000020 (p632_3)	NA	SWMV01000009 (p2470_1), SWMV01000043 (p2470_2)
Sequence Read Archive accession no.	PRJNA534061	PRJNA540014	PRJNA540012	PRJNA540010

a NA, not applicable.

The *S.* Heidelberg 329 and *S.* Typhimurium 2470 plasmids belong to the IncHI2 group and that of *S.* Heidelberg 632 to the IncII group. IncHI2 is a plasmid lineage associated with the spread of antibiotic-resistant *Salmonella* spp. (1). *S.* Heidelberg 329 and *S.* Typhimurium 2470 harbored a plasmid similar to *S.* Heidelberg N13-01290/pN13-01290_23 (GenBank accession number CP012931), isolated from turkey meat in Canada (14). A nucleotide BLAST search of specific contigs in *S.* Heidelberg 329 (p329_1) and *S.* Typhimurium 2470 (p2470_1) against the pN13-01290_23 sequence revealed 99% identity to both strain contigs with 81% coverage. *S.* Heidelberg pN13-01290_23 belongs to the IncHI2 group and carries *aph(3″)-Ib* and *aph(6)-Id* (aminoglycoside resistance), *bla*_TEM-1B_ (beta-lactam resistance), *sul1* (sulfonamide resistance), and *tet*(B) (tetracycline resistance) genes. *S.* Heidelberg 329 and *S.* Typhimurium 2470 have several antibiotic resistance genes, including *aac(6′)-Iaa*, *aph(3″)-Ib*, *aph(6)-Id, aadA1*, *bla*_TEM-1B_, *mph*(B) (macrolide resistance), *sul1* and *sul2* (sulfonamide resistance), *tet*(A) (tetracycline resistance), and *dfrA1* (trimethoprim resistance). All of these antibiotic resistance genes were located on the matching contig of pN13-01290_23, with the exception of the beta-lactam resistance gene, which was located in a different contig.

*S.* Heidelberg 632 and 2581 carry *aac(6′)-Iaa* (aminoglycoside resistance gene) and *fosA7* (fosfomycin resistance gene) only on their chromosomes. A previous study reported that the *fosA7* gene was found on the chromosome in *S.* Heidelberg isolated from poultry in Canada, although this gene is rarely reported among *Salmonella* spp. (15). Multidrug resistance efflux pump genes, such as *acrA* and *mdfA,* were detected on the chromosome in all *Salmonella* strains used in this study. Further understanding of antibiotic-resistant *Salmonella* spp. is required.

### Data availability.

The whole-genome shotgun project sequences of *Salmonella* strains 329, 632, 2581, and 2470 have been deposited at DDBJ/ENA/GenBank under the accession numbers listed in Table 1.
